# First Insights into the Intrapuparial Development of *Bactrocera dorsalis* (Hendel): Application in Predicting Emergence Time for Tephritid Fly Control

**DOI:** 10.3390/insects10090283

**Published:** 2019-09-03

**Authors:** Tian-Xing Jing, Ying-Xin Zhang, Wei Dou, Xin-Yi Jiang, Jin-Jun Wang

**Affiliations:** 1 Key Laboratory of Entomology and Pest Control Engineering, College of Plant Protection, Southwest University, Chongqing 400716, China; 2 State Cultivation Base of Crop Stress Biology for Southern Mountainous Land, Academy of Agricultural Sciences, Southwest University, Chongqing 400716, China

**Keywords:** *Bactrocera dorsalis*, emergence time, intrapuparial development, pest control

## Abstract

Intrapuparial development is a special pattern of metamorphosis in cyclorrhaphous flies, in which the pupa forms in an opaque, barrel-like puparium. This has been well studied in forensic insects for age estimations. In this study, the intrapuparial development of a quarantine agricultural pest, *Bactrocera dorsalis* (Hendel), was studied under a constant temperature of 27 ± 1 °C and 70 ± 5% relative humidity. Results showed that intrapuparial development could be divided into five stages: Larval-pupal apolysis, cryptocephalic pupa, phanerocephalic pupa, pharate adult, and emergent adult. It lays a morphology-based foundation for molecular mechanism studies and enhances the understanding of the physiological basis for changes in intrapuparial development. More importantly, the chronology of intrapuparial development can be used to predict the emergence time of tephritid flies, indicating when to spray insecticides to control these phytophagous agricultural pests. This may be an effective approach to reduce the use of insecticides and slow down the evolution of insecticidal resistance.

## 1. Introduction

The oriental fruit fly, *Bactrocera dorsalis* (Hendel) (Diptera: Tephritidae) causes substantial economic losses of commercial fruits and vegetables worldwide [1,2,3,4]. It is an invasive species and is listed as a quarantine pest in many countries. Chemical insecticides are the main control tools against this destructive pest. However, larvae are difficult to control because they are internal feeders inside fruits and thus are difficult to contact with insecticides [5]. The pupae, usually hidden in soil, are tolerant to environmental stresses, while the adult phase, especially when it has recently emerged, is the most vulnerable and best target [6]. Thus, the study of the chronological development of this fly is indispensable for predicting peak times for when flies eclose from puparia, which is the optimum time to spray insecticides.

Metamorphosis is found in nature among many organisms, including insects. The most metamorphosis in insects is holometabolism, where there are distinct egg, larval, pupal, and adult stages. In complete metamorphosis, the insect larva does not molt into the final adult form but a special form, the pupa. It is an effective strategy for insects to protect themselves against biotic and abiotic stresses. Cyclorrhaphous flies have unique pupae among insect species, owing to the opaque, barrel-like structure, called a puparium. This puparium is formed from the cuticle of the last larval instar (third-instar), and the pupa resides inside this puparium until eclosion.

Owing to the active roles of many flies in human life, this period from pupariation (puparia formation) to adult emergence is well studied in some cyclorrhaphous flies, especially necrophagous flies, such as *Chrysomya albiceps* [7] and *Sarcophaga dux* [8]. It is a useful tool to estimate the time of past events and predict future events by observing the pupal morphological changes. In practice, necrophagous flies’ pupae were used to accurately determine the flies’ age, which allows the determination of the minimum postmortem intervals (_min_PMI) in forensic studies. For predicting future events in fly life histories, changes in the compound eyes of fly pupae were used to predict the emergence of adults of the tephritid fly *Rhagoletis indifferens* [9]. The tephritidae family has many invasive flies, such as *B. dorsalis* (Hendel) and *Ceratitis capitata* [10], and is of importance to commercially produced fruits and vegetables. However, studies on the pupariation of tephritid flies, including those of economic importance, are limited. Pupal developments were described in several temperate tephritid flies, specifically *R. indifferens*, *Rhagoletis pomonella,* and *Rhagoletis cerasi* [9,11,12]. However, these three species have a diapause phase in the pupal stage (to varying degrees) and require some chilling for maximal adult eclosion. They are quite different from the non-diapause tropical fruit flies. Thus, optimal control of *B. dorsalis* requires more information regarding its biological features. This study was initiated to describe the chronology and metamorphosis of intrapuparial development events in *B. dorsalis*, which could be used in predicting the emergence time of this species.

## 2. Materials and Methods

The stock colony of *B. dorsalis*, originally obtained from a wild population in the Hainan province of China, have been reared in our laboratory for over 100 generations and were fed with an artificial diet [13]. This artificial diet mainly consisted of 45: 19: 15: 15: 6 parts of corn powder: Wheat powder: Yeast powder: Sugar: Agar, as well as a small amount of ascorbic acid and nipagin. Eggs were collected from *B. dorsalis* females by inducing oviposition through perforated parafilm coated with orange juice for 1 h, then placed on moist fabric and maintained at 27 ± 1 °C and 70 ± 5% relative humidity [14]. When the larvae emerged from the eggs, over 800 first-instar larvae were transferred to the artificial diet by using a brush, then the larvae were conditioned in a climate-controlled incubator at 27 ± 1°C and 70 ± 5% relative humidity with a 14 h photophase. The artificial diet was renewed daily to meet the nutritional requirements for larval growth. At the end of the third instar stage, when the larvae stopped eating and emptied their guts (the post-feeding larval or wandering stage), the insects were individually transferred into plastic glasses filled with sterilized sand to observe the pupariation process. When pupation was initiated, as indicated by the appearance of the sclerotic cuticle (white prepupa), the pupae were collected, fixed in 4% paraformaldehyde for 24 h and then preserved in 70% ethanol. The pupae collected at this time point were defined as 0 h pupa. In the next 48 h, pupae (actually prepupae) were collected every 2 h. After 48 h, pupae were collected every 6 h until adult emergence. 20 pupae were collected at every time point and were all dissected under a stereomicroscope and photographed with a digital microscope (Keyence VHX-5000, Osaka, Japan). For each specimen, photos of the ventral, dorsal, and lateral sides were taken. The process of head evagination was observed under the stereoscope (Optec SZ680T2L, Chongqing, China).

## 3. Results

### 3.1. Pupariation

After larvae stopped eating and emptied their guts, they exited the diet and crawled into the sand, where they became immobile. During the last hour of this post-feeding periods, the larvae gradually became inactive and longitudinally shortened themselves. This shortening resulted in a smooth cuticle and increased diameter from the elimination of constrictions between segments.

### 3.2. Larval-Pupal Apolysis (0–34 h, 0–1.42 days, 32.4 ± 0.23 h)

**Figure 1:**
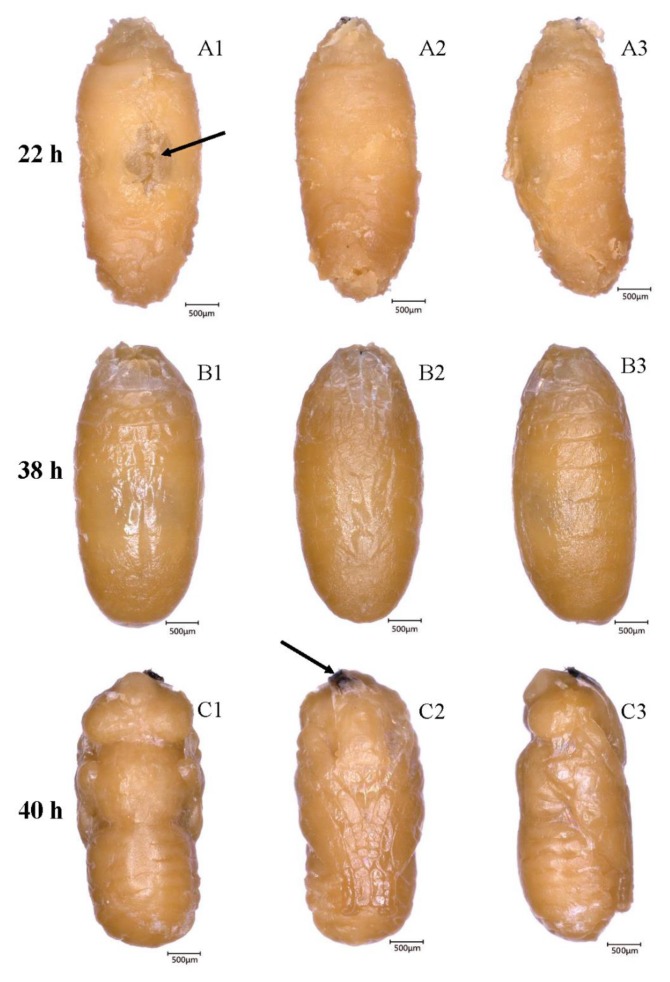
Intrapuparial development of *B. dorsalis* in the first 48 h. (**A1**–**A3**) Dorsal, ventral, and lateral morphology of the larval-pupal apolysis stage, the arrow shows the bubble (22 h); (**B1**–**B3**) dorsal, ventral, and lateral morphology of the cryptocephalic pupa (38 h); (**C1**–**C3**) dorsal, ventral, and lateral morphology of the phanerocephalic pupa; the arrow shows the shed cephalo-pharyngeal apparatus (40 h).

**Figure 2:**
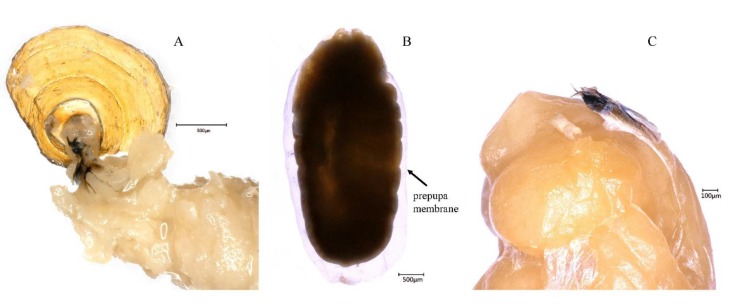
Details of *B. dorsalis* during larval-pupal apolysis to the phanerocephalic pupa. (**A**) The cephalo-pharyngeal apparatus remained attached to the puparium in the larval-pupal stage; (**B**) the prepupa membrane was shed after larval-pupal apolysis; (**C**) the cephalo-pharyngeal apparatus on the anterior ventral portion of the phanerocephalic pupa.

This apolysis phase ranged from 0 h to 34 h, 0–1.42 days after puparium formation (Figure 1), and the mean value of this phase with SE was 32.4 ± 0.23 h (shown in the brackets in subtitle). The puparium of the prepupa became progressively more opaque, pigmented, and sclerotized. At the beginning of this stage, the hypodermis was firmly attached to the puparium, and it was difficult to separate the hypodermis from the puparium. During apolysis, the epidermis gradually separated from the cuticle. In this stage, the cephalo-pharyngeal apparatus hidden in the segment was attached to the puparium (Figure 2A). Additionally, a cavity, full of air and looking like a bubble, appeared in the middle of the dorsal area. The bubble increased in volume, forcing the tissue between the bubble and puparium to become very thin. At the end of apolysis, the bubble disappeared. The term “prepupa” should be used to describe the insect during the larval-pupal apolysis period.

### 3.3. Cryptocephalic Pupa (32–40 h, 1.33–1.67 days, 4.7 ± 0.22 h)

When the larval-pupal apolysis stage was completed, the term “pupa” should be used to describe the insect, although the pupa at first remains in a larval shape. In the first pupal stage (cryptocephalic pupa), the cephalo-pharyngeal apparatus, was still hidden inside the insect segments. After ~2 h, the thoracic segments were formed and the imaginal appendages (legs and wings) on the thoracic area were observed as the thoracic segments differentiated. The appendages surrounded by the delicate prepupal cuticle were now considerably short and extended to the fourth abdominal segment. However, the head was still hidden in the thorax. During this stage, visible active peristalsis under the prepupal membrane was observed.

### 3.4. Phanerocephalic Pupa (36–42 h, 1.5–1.75 days, 5.5 ± 0.20 h)

At 36–42 h after pupariation, the abdominal muscles contracted and forced the hidden head to evaginate. This evagination progress happened in a very short time period (several seconds), and fat body-like tissues were pumped into the newly formed head at the time of head evagination. Three sections of the pupa-the head, thorax, and abdomen-could now be distinguished. After this event, the stomodeum, proctodeum, and posterior trachea were shed from the puparia. Additionally, the cephalo-pharyngeal apparatus was shed and found at the ventral portion of prepupal cuticle (Figure 2C).

### 3.5. Pharate Adult (66–228 h, 2.75–9.5 days, 133.2 ± 1.55 h)

**Figure 3:**
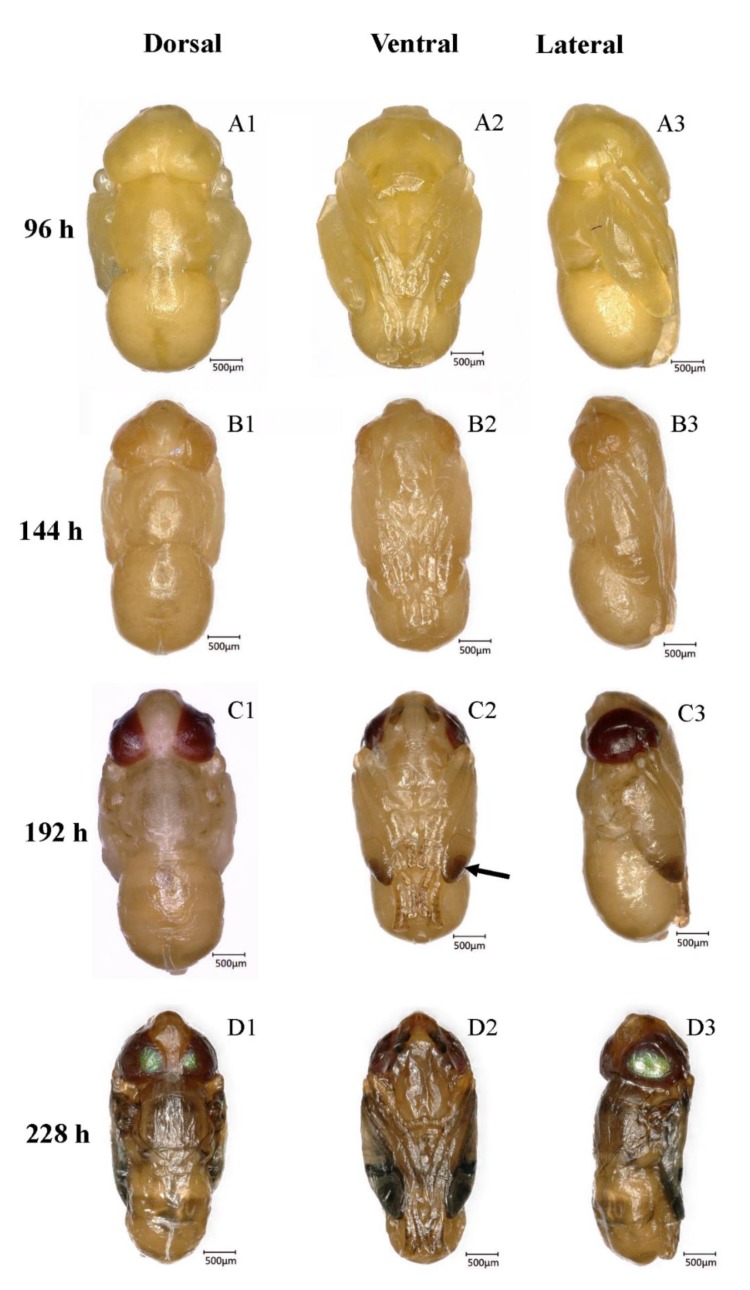
Intrapuparial development of *B. dorsalis* pharate adult. (**A1**–**A3**) Dorsal, ventral, and lateral morphology of the transparent-eyed pupa (96 h, 4 days); (**B1**–**B3**) dorsal, ventral, and lateral morphology of the yellow-eyed pupa (144 h, 6 days); (**C1**–**C3**) dorsal, ventral, and lateral morphology of the reddish brown-eyed pupa, the arrow shows the tanned wing tin (192 h, 8 days); (**D1**–**D3**) dorsal, ventral, and lateral morphology of the metallic red-eyed pupa (228 h, 9.5 days).

This is the period during which the pupa matures into an adult, and it is the longest stage in intrapuparial development. The period lasts from the end of pupal-adult apolysis to adult emergence. This stage can be divided into four sub-stages on the basis of the compound eye pigmentation (Figure 3).

#### 3.5.1. Transparent-Eyed Pupa (66–138 h, 2.75–5.75 days, 59.7 ± 1.02 h)

During this stage, the cuticle of the whole body and the compound eyes were soft and white. Bristles on the head near the compound eyes and on the thorax formed at ~120 h. These bristles were found on the surface of the imaginal cuticle under the pupal cuticle, and they were colorless at the early stage. Under the cuticle, the fat body existed in the form of globules. The evaginable appendages elongated rapidly in length, and the legs finally reached the posterior end of the abdomen. Leg segments, such as the tibiae and tarsus, could be observed easily. The wings were full of semi-liquid fat bodies, resembling flattened bags. Additionally, a projection that would finally develop into the ovipositor was found in the appropriate position in the female adult. This means that sexual identification of flies can be done in the pupal stage.

#### 3.5.2. Yellow-Eyed Pupa (144–180 h, 6.0–7.5 days, 34.2 ± 0.63 h)

**Figure 4:**
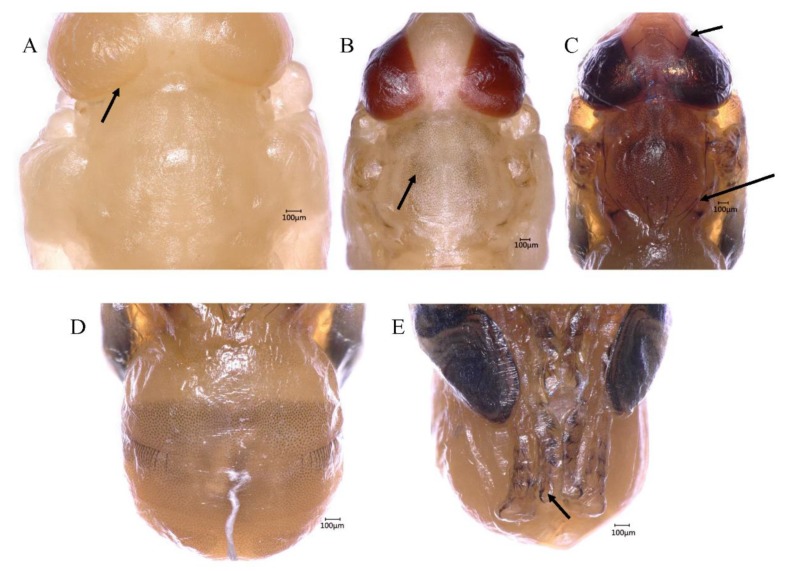
Details of *B. dorsalis* pharate adult. (**A**) View of the yellow-eyed pupa, the arrow shows the boundary; (**B**) black spots on the dorsal thorax; (**C**) the arrow shows the black bristles on the head and thorax; (**D**) black spots on the dorsal abdomen; (**E**) the arrow shows the sclerotization of legs.

The color of the body was still white, but the compound eyes were yellow, and this stage was defined because of the color and the clear boundaries of compound eyes (Figure 4A). The bristles were still transparent. Overall, only the difference in eye color marks these stages.

#### 3.5.3. Reddish Brown-Eyed Pupa (186–222 h, 7.75–9.25 days, 33.9 ± 0.66 h)

Reddish brown compound eyes appeared along with the black spots on the dorsal thorax and abdomen, but most of the body remained white, with a little brown. The visible horizontal stripes on the second segment, formed by these black spots, appeared first and were followed by bands on other abdominal segments (Figure 3C). The bristles were gradually pigmented. In addition to the change in eye color, the antennae, wing tips, and legs became tanned with time (Figure 3C). Finally, the wings became thinner and the folded structure formed, with some veins being visible. The legs, to a certain extent, were sclerotized, and the claws on the leg tips appeared.

#### 3.5.4. Metallic Red-Eyed Pupa (222–228 h, 9.25–9.5 days, 5.4 ± 0.41 h)

With pupal development, the eyes and the facial region became fully formed and the compound eyes became metallic red. The wings, legs, and dorsal thorax were fully pigmented. The wings became much thinner and quite similar to those of a newly emerged fly.

### 3.6. Emergent adult (228–246 h, 9.5–10.25 days, 9.6 ± 0.91 h)

At the end of the longest period, the pharate adult flies emerged from the puparia (228–246 h, 9.5–10.25 days). The newly emerged fly had a relative whitish abdomen and completed pigmentation in 15 min. The folded wings stretched and hardened in 20 min.

## 4. Discussion

More detailed knowledge on the invasive pest *B. dorsalis* will contribute to developing effective strategies for its control and quarantine issues [15,16]. Although various pest management techniques have been employed [17,18,19,20], controlling this fly has mainly relied on spraying insecticides, and insecticides resistance in *B. dorsalis* has been reported in many countries [16]. Therefore, a reasonable method or strategy is necessary to reduce resistance that results from the overuse or improper use of insecticides. In tephritid species, females lay eggs by piercing the fruit or vegetable skin. The larvae feed on nutrients in the fruit pulp, and the fruit protects larvae from enemies and insecticides [5]. This feeding trait makes the larval stage a difficult target period for control strategies. Unfortunately, the pupa is also not a target stage for insecticides because of the larvae pupate in the soil. In this pupal stage, the soil and hard puparia act as barriers against insecticides. Overall, the adult stage, especially upon emergence, is the appropriate target period to control the fruit flies. Therefore, predicting the emergence time would allow the optimum use of insecticides or attractants.

Using insect morphological changes to estimate the occurrence time of an event is a popular method in forensic entomology. Intrapuparial chronology of many saprophagous flies, such as *Lucilia sericata* [21], *Cochliomyia macellaria,* and *Lucilia cuprina* [22] were studied and used in calculating the minimum postmortem intervals (_min_PMI). The larval development is of equal importance in forensic cases [23]. There was no stage between egg laying and the larval stage, and the interval time was relatively short. Thus, using the larval chronology to calculate the _min_PMI was more accurate than observing the pupae in the blow fly.

However, we noticed that the larval stage is not an appropriate period for predicting the emergence time of tephritid flies, such as *B. dorsalis*, because the interval time between the larval stage and emergence (~20 d in *B. dorsalis*, including the intrapuparial stage) was too long to predict the emergence time accurately. Moreover, the larval size (most often length) used to estimate larvae age was variable (even they reduce their lengths at the termination of the third-instar stage), and this variation complicated the estimation process [23]. Additionally, our previous study reported that body length is not a key feature for accurately determining the instars of *B. dorsalis*, because the larval lengths amplitudes overlapped between adjacent instars [14]. Therefore, we focused on the intrapuparial stage and used pupal chronology to predict the emergence time. In fields, it is not easy to sample pupae in soil, but it is easy to collect amounts of larvae in infested fruits. When these collected larvae pupate, we can bury them in soil in field and the buried places can be flagged. This may help us to obtain pupae used in prediction of emergence time.

At present, almost all the intrapuparial studies have focused on the saprophagoous insects used as forensic tools. Among phytophagous flies, the intrapuparial development of several model species were clear. As the subject of genetic and physiological research, the metamorphosis of *Drosophila melanogaster* has been described [24,25]. The intrapuparial development of *Delia antiqua,* as an ideal model for research on insect pupal diapause, was observed [26]. In Tephritidae, several temperate flies’ intrapuparial development were studied, such as *R. indifferens*, *R. pomonella*, and *R. cerasi* [9,11,12]. However, these three species experience a diapause in the pupal stage and their pupal stages last over 10 months. They are quite different from the non-diapause tropical fruit flies, *C. capitata*, *Anastrepha obliqua*, and *B. dorsalis* [27,28,29]. Although there was limited knowledge on phytophagous flies, the features of intrapuparial development are relatively conservative in non-diapause flies [30]. In this study, the intrapuparial development of *B. dorsalis* was described, and the development was divided into larval-pupal apolysis, cryptocephalic pupa, phanerocephalic pupa, pharate adult, and emergent adult. The process from larval-pupal apolysis to adult emergence, at a constant 27 °C, lasted ~246 h. Furthermore, we compared the intrapuparial development of *B. dorsalis* with that of some other non-diapause cyclorrhaphous flies (Table 1). Most of the intrapuparial development of non-diapause flies can be divided into the same five phases as *B. dorsalis,* but the duration is quite different. The time duration of the intrapupal stage in *Sarcophaga dux* (240 h, 22 °C) was similar as *B. dorsalis*, while it was shorter (76 h, 25 °C) in *D. melanogaster* and longer (~348 h) in *Sarcophaga (Neobellieria) bullata* Parker at 24 °C. In *Anastrepha ludens*, the intrapuparial stage was divided as six successive phases; coarctate larva, cryptocephalic pupa, phanerocephalic pupa, early pharate adult, mid-pahrate adult, and late pharate adult [31]. Although differences in the duration and terminology of each phase existed among species, the landmarks were consistent.

In diapause flies, the intrapuparial period can also be divided into the same five phases like non-diapause flies, and the diapause often occurs after cryptocephalic and before the pharate adult phase [12,26]. In *R. pomonella,* the description of pupal development was earlier, resolving confusion of terminology in intrapuparial development, so the terminology of the phases used in that study was different from this study. It was described as the cryptocephalic (2–5 days), phanerocephalic (2 days over winter), teleomorphic (2–30 days before first emergence), chromoptic (2–16 days before first emergence), and chromogenic (2–7 days before first emergence) phase. Although the terms used in *R. pomonella* were different, the changes of morphological features were similar, such as the head evagination in the phanerocephalic phase and the compound eye color changes in the chromoptic phase. These obvious changes in *R. pomonella* can also be used in predicting fly emergence: Approximately 1 month (teleomorphic phase) and 1 week (chromogenic phase) before emergence [11]. Similarly, it is also reported that the emergence date of *R. indifferens* can be predicted approximately 1 week before by noting the change in its compound eyes [9].

For flies, environmental temperature has a great influence on the timing of intrapuparial development [33]. The larval-pupal apolysis of *Sarcophaga argyrostoma* is completed 30–32 h after pupariation at 29 °C. However, the time duration of larval-pupal apolysis is 36–40 h at 25 °C. A similar phenomenon is found in *C. albiceps*, even though the larval-pupal apolysis stage was shorter and the pharate adult stage was longer at 28 °C in the day/26 °C at night than at a constant 26 °C [34]. In most cases, cooler temperatures slowed down development, while warmer temperatures had the opposite effect. Thus, the temperature in the field is an indispensable parameter. Additionally, as demonstrated in forensic entomology, the type of tissue used to feed blow flies impacts the insect size and developmental rate of both larvae and pupae. In some fly species, the diet provided has a greater impact on growth (in both larval and pupal stages) than the temperature [35]. In the tephritid fly, *R. pomonella*, host plants affect the initial pupal diapause depth and developmental rate in the post-diapause period [36]. As a polyphagous pest, *B. dorsalis* can damage many kinds of fruit, including those of *Psidium guajava*, *Mangifera indica,* and most citrus [1]. It is likely that various host plants may have different effects on the duration of *B. dorsalis* pupae and the estimation of emergence time. Therefore, the host plant of flies is also a factor that should be considered. In addition, the genetic background can also affect the accuracy of the estimation of emergence time [37,38], and more research is needed to understand how genotypes of the same species affect development.

Entomologists have tried to use molecular biotechniques to estimate the development of the intrapuparial stage [39]. In Calliphoridae, using the gene expression level changes during the intrapuparial stage is an effective way to determine age. In theory, the gene chosen for indicating the intrapuparial stage should have an expression level that corresponds to the specific time point during pupal development [40]. This means that the quantitative marker genes should have continuously increasing or decreasing expression levels during the intrapuparial period. However, marker gene identification is still progressing and few genes can meet the above criteria. Previous studies focused on screening time-dependent genes, such as *actin* and *15_2*, which are related to cell growth, cell death, metamorphosis, or other developmental process [41,42]. In addition, another new technique, micro-computed tomography (micro-CT) has been used to study intrapuparial development in recent years. Richards et al. (2012) first used this new method to identify fly age during the intrapuparial stage, and this method has become more systematic with use [43]. This non-destructive method can be used to qualitatively and quantitatively analyze organ changes [44]. Unlike the traditional dissection-based analysis of puparium, micro-CT focuses on morphological changes of the internal organs [45,46]. The wealth of information provided by micro-CT could resolve problems associated with misuses and misinterpretations [46,47]. However, micro-CT is not routinely used because it is new and not available to most entomologists. It is certainly a suitable way to improve the precision and accuracy of age estimations when used in combination with external morphological observations and gene expression analyses.

## 5. Conclusions

In conclusion, the intrapuparial development of *B. dorsalis* was divided into larval-pupal apolysis, cryptocephalic pupa, phanerocephalic pupa, pharate adult, and emergent adult phases. Based on the intrapuparial development of *B. dorsalis*, we put forward a new method to predict the emergence time of fruit flies. It is a common tool used in forensic entomology, and, from the experience in forensic entomology, it is a feasible method to estimate the pupal age and emergence time, which is crucial to determine the time of spraying insecticides. Although it takes time to put this method into practice, the key parameters, such as temperature and food source, are well studied in forensic entomology and will be considered in further studies.

## Figures and Tables

**Table 1:** Comparison between the time of the intrapuparial development of *B. dorsalis* with that of other non-diapause flies. Larval-pupal apolysis (LPA), cryptocephalic pupa (CCP), phanerocephalic pupa (PCP), pharate adult (PHA), emergent adult (EAD), references (REF).

Development Stages (hours)							
Species	Temperature (°C)	LPA	CCP	PCP	PHA	EAD	REF
*Drosophila melanogaster*	25	4	4–6	12	24	76	[25]
*Musca domestica*	30	4	4–9	16–18	28–30	96–110	[30]
*Lucilia sericata*	25	11	11–21	22–47	69–167	175	[21]
*Sarcophaga dux*	22	24	24	48	192	240	[8]
*Sarcophaga (Neobellieria) bullata* Parker	24	20	20–28	46–48	168–192	324–348	[30]
*Chrysomya albiceps*	28	0–4	4–6	6–10	24–96	99	[7]
*Cochliomyia macellaria*	23	0–27	6–42	12–78	15–90	120	[32]
*Lucilia cuprina*	23	0–36	6–39	15–34	18–192	210	[22]
*Ceratitis capitata*	23	0–40	40–46	46–48	48–264	288	[27,28]
*Bactrocera dorsalis*	27	0–34	32–40	36–42	66–228	228–246	This study
